# Screening of cytochrome 4Z1 expression in human non-neoplastic, pre-neoplastic and neoplastic tissues

**DOI:** 10.3332/ecancer.2020.1114

**Published:** 2020-09-29

**Authors:** Yousef M Al-saraireh, Nafea S Alboaisa, Hamzeh Mohammad Alrawashdeh, Omar Hamdan, Sameeh Al-Sarayreh, Jehad M Al-Shuneigat, Mohammad N Nofal

**Affiliations:** 1 Department of Pharmacology, Faculty of Medicine, University of Mutah, Karak 61710, Jordan; 2 Department of Pathology, College of Medicine, University of Anbar, Baghdad 55431, 55 Ramadi; 3 Department of Ophthalmology, Ibn Al Haytham Hospital, Amman 410739, Jordan; 4 Department of Pathology, College of Medicine, University of Mutah, Karak 61710, Jordan; 5 Department of Biochemistry and Molecular Biology, Faculty of Medicine, University of Mutah, Karak 61710, Jordan; 6 Department of General Surgery, Faculty of Medicine, University of Mutah, Karak 61710, Jordan

**Keywords:** cancer, breast cancer, cytochrome P450, CYP4Z1, immunohistochemistry

## Abstract

**Background:**

Cytochromes P450 (CYPs) constitute an enzyme family involved in the oxidative metabolism of a wide variety of endogenous and exogenous compounds, including anti-cancer drugs and carcinogens. Unlike other human CYPs, CYP4Z1 is highly expressed in human breast carcinoma and is associated with poor prognosis. As a result, CYP4Z1 was hypothesised to be a potential biomarker or drug target for the discovery and development of promising anti-cancer therapies.

**Materials and methods:**

CYP4Z1 expression was immunohistochemically studied in a set of 100 different human tissues, including normal, benign, malignant and metastatic tissues, which originated from 27 anatomical sites. As a tumour model for CYP4Z1 expression, a panel of different breast cancers was evaluated for CYP4Z1 expression and its relation to histopathological features and prognostic immunohistochemical markers.

**Results:**

The immunohistochemical results revealed that CYP4Z1 was expressed in only one (4.3%) of the normal tissues from the mammary glands, while the expression of the enzyme was positive in 1 (11%), 12 (19%) and 2 (40%) of the benign, malignant and metastatic tissues, respectively. Interestingly, several tumour entities showed prominent expressions of CYP4Z1, including carcinomas of adrenal cortex, squamous cells of oesophagus, lung and cervix, as well as seminoma, astrocytoma, melanoma and lastly endometrial adenocarcinoma. In breast cancers, CYP4Z1 was expressed in 82% of the cases. Its expression was significantly associated with the pathology of tumour, histological grade and status of lymph node metastasis. Importantly, it was also significantly associated with the expressions of Her2, P53 and Ki-67.

**Conclusion:**

These findings greatly support future plans for the use of CYP4Z1 as a biomarker or target for anti-cancer drugs. However, large-scale validation studies are needed to better delineate the potential use of CYP4Z1 for therapeutic purposes.

## Introduction

Cytochromes P450 (CYPs) are a large superfamily of membrane-bound monooxygenase heme-containing enzymes found in all domains of life. They catalyse a variety of complex reactions, predominantly mixed-function oxidations, often displaying highly regioselective and/or stereoselective chemistry [1]. They play vital roles in endogenous biosynthetic pathways, including those of sterols, fatty acids, prostaglandins, vitamins and hormones, and, in addition, are involved in the metabolism of xenobiotic compounds, such as antibiotics and environmental toxins [2, 3], and in the breakdown of aromatic petrochemicals [4]. They catalyse a variety of NADPH/NADH- and O_2_-dependent reactions, including hydroxylation, alcohol to carbonyl oxidation; epoxidation; dealkylation; heteroatom oxidation; desaturation; ring cleavage, expansion, coupling and formation; dehydration and even reduction [5].

Based on the catalytic activity, CYPs are classified into xenobiotic-metabolising enzymes, including CYP1, CYP2, CYP3 and CYP4, and endogenous substrate-metabolising enzymes, including CYP11, CYP17, CYP19 and CYP21 [6]. Therefore, CYP systems fulfil diverse functions and are implicated in many biological processes such as carcinogenesis and response to therapeutic drugs, as well as cell signalling and regulation [7].

There is a significant level of interest in the role and functions of CYPs in tumour formation and progression. Many studies show that some individual forms of CYPs are selectively overexpressed in some tumours compared to the adjacent normal tissues. Moreover, the expression of these specific enzymes may also show tumour-type selectivity [6]. The overexpression of the individual forms of CYP are determined in many tumours, including cancers of the lung, colon, breast, oesophagus, stomach, prostate, bladder, kidney and soft tissue sarcomas. Additionally, some of these enzymes participate in the fate of anti-cancer drugs as taxans, which are key agents in the treatment of tumours [8]. Therefore, this system of enzymes may represent a useful and promising anti-cancer therapeutic target. Several therapeutic strategies are currently under development, based on CYP targets, including modification of the rate of CYP expression, CYP-mediated pro-drug activation and CYP inhibitors [9].

One such possibility that has attracted considerable interest in tumour development and therapy is the only member of the CYP4Z subfamily (CYP4Z1). It is an extrahepatic enzyme with a poorly defined substrate spectrum and biological function [10]. This enzyme is preferentially expressed in mammary tissues with undetectable levels in other normal human tissues. It was found to be overexpressed in different types of tumours as primary breast cancer tissues compared to their corresponding normal tissues [11, 12]. Moreover, this expression was clarified to be inducible in glucocorticoids and progesterone-treated breast cancer cells [13]. In profiling the expression of CYP enzymes in breast, ovary and colon cancers, CYP4Z1 was overexpressed in all tumours examined compared to normal tissues, and its expression in ovary cancer was suggested to be a biomarker for prognosis [12, 14–16]. Moreover, the elevated CYP4Z1 expression was correlated with a higher mortality rate in patients with prostate cancer and was suggested as an independent prognostic marker for prostate cancer [17].

Several functional studies investigating the catalytic activity of CYP4Z1 have been reported. CYP4Z1 was found to catalyse ω-hydroxylation of saturated mid-chain fatty acids. Different monohydroxylated metabolites of lauric acid and myristic acid were detected at several positions [18, 19]. Moreover, high levels of 20-hydroxyeicosatetraenoic acid (20-HETE) metabolites were possibly generated through the metabolism of arachidonic acid by CYP4Z1 [19]. Conversely, a recent functional analysis reported the absence of 20-HETE metabolites in the CYP4Z1 reaction with arachidonic acid in a recombinant enzyme system [20]. This discrepancy needs further study with purified reconstituted CYP4Z1 to examine the enzyme’s substrate and product specificities.

On validating CYP4Z1 as a potential biomarker or drug target for anti-cancer drug development, the aims of this study were firstly to analyse immunohistochemically the expression of CYP4Z1 in different human tissues, including normal, benign, malignant and metastatic tissues, which originated from 27 anatomical sites. Secondly, this study aims to characterise CYP4Z1 expression in different types of breast cancer and to explore the relationship between the expression of CYP4Z1 and histopathological features, as well as key prognostic immunohistochemical markers used for breast cancers.

## Materials and methods

### Tissue specimens

All patients’ specimens used in this study were formalin-fixed, paraffin-embedded tissues (4-μm thick). These were obtained from the pathology department, King Hussein Medical Hospital, Royal Medical Services, Amman and King Abdullah University Hospital, Irbed, Jordan. All personal data associated with the specimens were kept anonymous. The study was ethically approved by the Ethics Committee, Faculty of Medicine, Mutah University, Jordan

### Multi-tumour tissue samples

A set of 100 patient samples, which originated from 27 different anatomical sites, was used for screening. Of them, 77 were from 40 tumour entities and the rest were normal tissue specimens from different human organs.

### Breast carcinogenesis tissue samples

Based on the results obtained from the multi-tumour set of different human tissues, the expression of CYP4Z1 was further assessed in a panel of human breast cancers. This panel consisted of eight normal breast tissues and 220 breast cancer types. All samples were formalin-fixed, paraffin-embedded tissues. The patients’ data were retrospectively obtained from patient records and who were referred to King Hussein Medical Hospital, Royal Medical Services and King Abdullah University Hospital, Amman and Irbed, Jordan. Patients who underwent preoperative chemotherapy or radiotherapy were excluded from the study. The data regarding biomarkers expression (AR, epidermal growth factor receptor (EGFR), oestrogen receptor (ER), progesterone receptor (PR), epidermal growth factor receptor 2 (HER-2), P53 and Ki67) and clinicopathologic features, including age, histological type, histological grade, histological stage and lymph node metastasis, were extracted from patient files.

### Immunohistochemistry

The obtained tissue sections for the present study were dewaxed in xylene and rehydrated to water through an alcohol gradient. Endogenous peroxidase activity was quenched with freshly prepared 3% hydrogen peroxide for 5 minutes at room temperature. Sections were then subjected to heat-induced epitope retrieval in 10 mM citrate buffer, pH 6.0, in a microwave at 600 W for 20 minutes. 1.5% normal goat serum was used to block the non-specific binding sites for the antibodies in the studied sections for 20 minutes at room temperature in a humidified atmosphere. The sections were then incubated with a primary rabbit monoclonal antibody specific for anti-CYP4Z1 (NBP1-91817) (Novus biological, USA) at a concentration of 2 μg/ml for 1 hour at room temperature. The specificity of the given antibody towards CYP4Z1 protein was tested using CYP4Z1 isogenic cell lysates by Western blotting (data not shown). Following the primary antibody treatment, sections were incubated with goat anti-rabbit peroxidase-conjugated secondary antibody (Vector Laboratories Ltd, Peterborough, UK) 7.5 μg/ml concentration for 30 minutes at room temperature, then washed twice in phosphate buffered saline, pH 7.4. The colour reaction was developed by incubating the treated sections with 3, 3-diaminobenzidine chromogen solution (Vector Laboratories Ltd, Peterborough, UK) for 3–5 minutes. The sections were then counterstained in Harris’s haematoxylin solution mounted with coverslips using dibutylphthalate polystyrene xylene (DPX) medium. The negative control samples (procedural controls) were prepared by omitting the primary antibody in order to examine the secondary antibody binding.

The resulting slides were viewed and analysed using a Leica DMRB microscope (Leica DMRB, Wetzlar, Germany) with the images digitally captured and processed using a Leica MPS52 camera (Q Imaging, Germany) and the AcQuis imaging capture system (Synoptics, Cambridge, UK), respectively.

### Analysis of CYP4Z1 expression

Based on the previously described semi-quantitative methods [21], evaluation of the expression of CYP4Z1 was manually carried out by two independent pathologists. The cells were considered positive for CYP4Z1 expression if they demonstrated clear membranous or cytoplasmic immunoreactivity. The scoring results were presented as none (0), weak (1), moderate (2) and high (3). The score ‘none’ indicated an absence of expression. A score of ‘weak’ was applied to cells that had an expression of less than 33%. Sections were allocated a score of ‘moderate’ when they had an expression of 33%–66% of the cells, whilst the score of ‘high’ indicated an expression in more than 67% of the cells.

### Statistical analysis

Statistical analysis of data was carried out using the available statistical package of SPSS-25 (Statistical Packages for Social Sciences, version 25). Data were presented in simple measures of frequency and percentage. The differences between the discrete variables were evaluated by Pearson’s Chi-square test (2-test) with application of Yate’s correction or Fisher’s exact test whenever applicable. The results were considered statistically significant only if *p* < 0.05.

## Results

### CYP4Z1 expression in normal tissues

**Figure 1.**
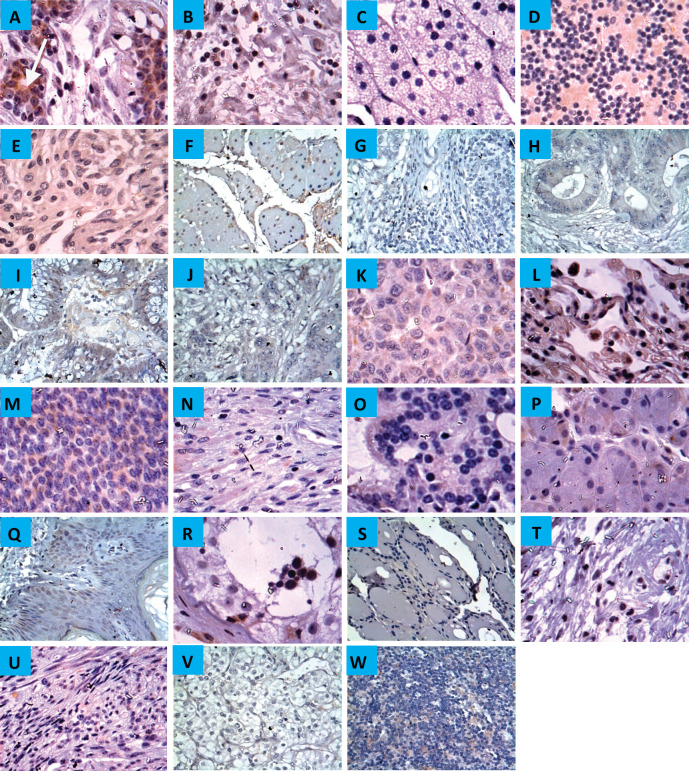
Expression of CYP4Z1 in different normal tissues. (A) Distinct CYP4Z1 labelling can be seen in the cell cytoplasm in breast tissues. A negative CYP4Z1 expression was seen in (B) bladder, (C) adrenal gland, (D) cerebellum, (E) cerebrum, (F) oesophagus, (G) stomach, (H) intestine, (I) colon, (J) rectum, (K) liver, (L) lung, (M) ovary, (N) pancreas, (O) prostate, (P) salivary gland, (Q) skin, (R) testis, (S) thyroid, (T) cervix, (U) uterine endometrium, (V) renal cortex and (W) lymph node. Magnification (×400).

Immunoreactivity for CYP4Z1 was predominantly localised to the membrane or cytoplasm of cells without any significant staining noted in the nucleus. In normal tissue specimens, only one of the breast specimens was stained positively for CYP4Z1 (4.3%). This staining was characterised with weak immunoreactivity. Despite background staining seen in some tissues, no CYP4Z1 immunoreactivity was found in the following types of tissues: adrenal gland, bladder, colon, cerebellum, cerebra, renal cortex, liver, lung, lymph node, oesophagus, ovary, prostate, rectum, stomach, small intestine, pancreas, salivary gland, testis, thyroid, skin, uterine endometrium and uterine cervix (Figure 1).

### CYP4Z1 expression in pre-neoplastic, neoplastic and metastatic tissues

**Figure 2.**
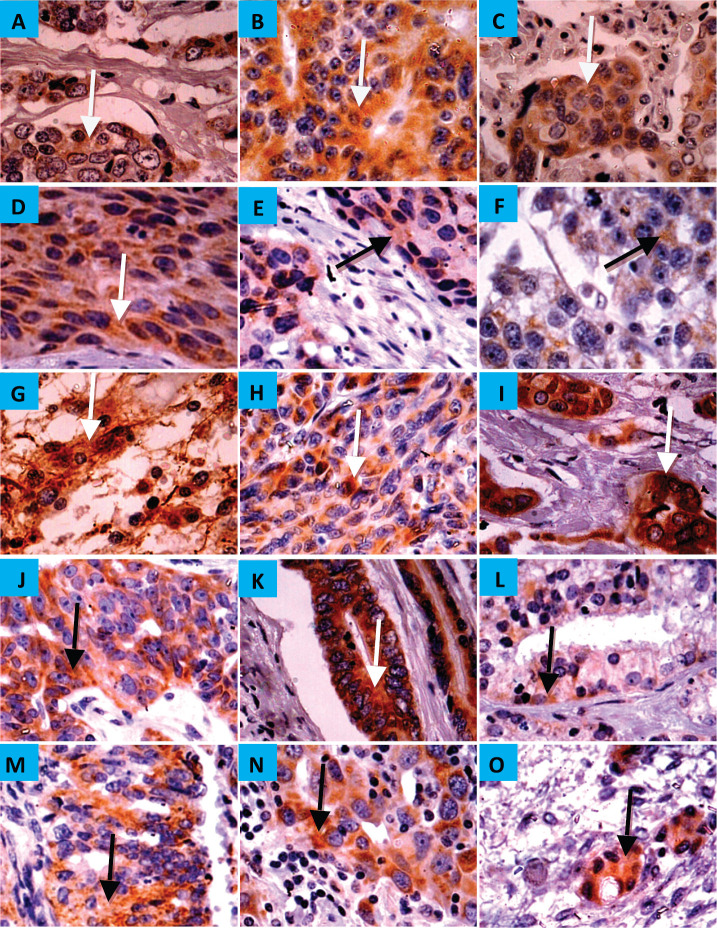
Expression of CYP4Z1 in benign, malignant and metastatic tumours. (A) Distinct CYP4Z1 labelling can be seen in the cell cytoplasm in breast adenoma. Several malignant primary tumours displayed CYP4Z1 expression, including carcinomas of adrenal cortex (B), squamous cells of oesophagus, lung and cervix (C, D and E, respectively), seminoma (F), astrocytoma (G), melanoma (H) and lastly adenocarcinomas of breast, ovary, colon, prostate and endometrium (I, J, K, L and M, respectively). CYP4Z1 immunoreactivity was also demonstrated in metastatic tissues of the lymph node (N) and ovary (O) that have been originally derived from primary carcinomas of breast and colon, respectively. Magnification (×400).

CYP4Z1 was expressed in 1 (11%), 12 (19%) and 2 (40%) of the benign, malignant and metastatic tissues, respectively. There was diffuse immunoreactivity with no evidence of intra-tumour heterogeneity of immunohistochemical staining. In benign tumours, only breast adenoma showed weak immunoreactivity for CYP4Z1, while it was negative for the adenomas of adrenal gland, small intestine, colon, salivary gland, ovary and thyroid. As expected, all specimens of breast adenocarcinoma revealed the most frequent expression of CYP4Z1. Nevertheless, several types of tumours demonstrated CYP4Z1 expression, including carcinomas of adrenal cortex, squamous cells of oesophagus, lung and cervix, astrocytoma, melanoma and seminoma, as well as adenocarcinomas of colon, ovary, prostate and endometrium (Figure 2). CYP4Z1 expression was also examined in several metastatic tissue specimens, such as liver, lung, ovary and lymph nodes, which were originally derived from primary carcinomas of colon, stomach, breast and squamous cell of lymph node, respectively. The immunoreactivity for CYP4Z1 was shown to be positive in metastatic tissues of lymph node and ovary that were originally derived from primary carcinomas of breast and colon, respectively (Figure 2, panels N and O).

### CYP4Z1 expression in breast carcinogenesis tissue samples

#### Baseline demographic and clinicopathologic features for breast cancer patients

All demographic and clinical data were available for 228 females, where eight of them presented with normal pathology and 220 were diagnosed with breast cancer (Table 1). The mean age for patients was estimated at 48.7 ± 11.5 years. The most common pathology of breast cancer enrolled in this study was invasive ductal carcinomas (178 cases, 80.8%). Other pathologies included were 14 intra-ductal carcinomas (6.4%), 13 invasive lobular carcinomas (5.9%), six mucinous adenocarcinomas (2.7%), two invasive papillary carcinomas (1.0%), two lobular carcinoma *in situ* (1.0%), two invasive apocrine carcinoma and the remaining three cases (1.5%) were Paget’s disease, lipid-rich carcinoma and undifferentiated carcinoma, respectively. More than half of the breast tumours were of histological grade III (119 cases, 52.3%), whereas grade II constituted 40.7% (93 cases) and grade I 7% (16 cases). Regarding the histological stage, most of the cases were at stage 2 (144 cases, 63.2%), while 38 cases (16.7 %) were at stage 1 and 37 cases (16.2%) were at stage 3. Lymph node metastasis was detected in 61 cases (26.8%) and the remaining 158 cases had no lymph node metastasis (69.3%).

#### Prevalence of CYP4Z1 expression and its relation to demographic and clinicopathologic features

**Figure 3.**
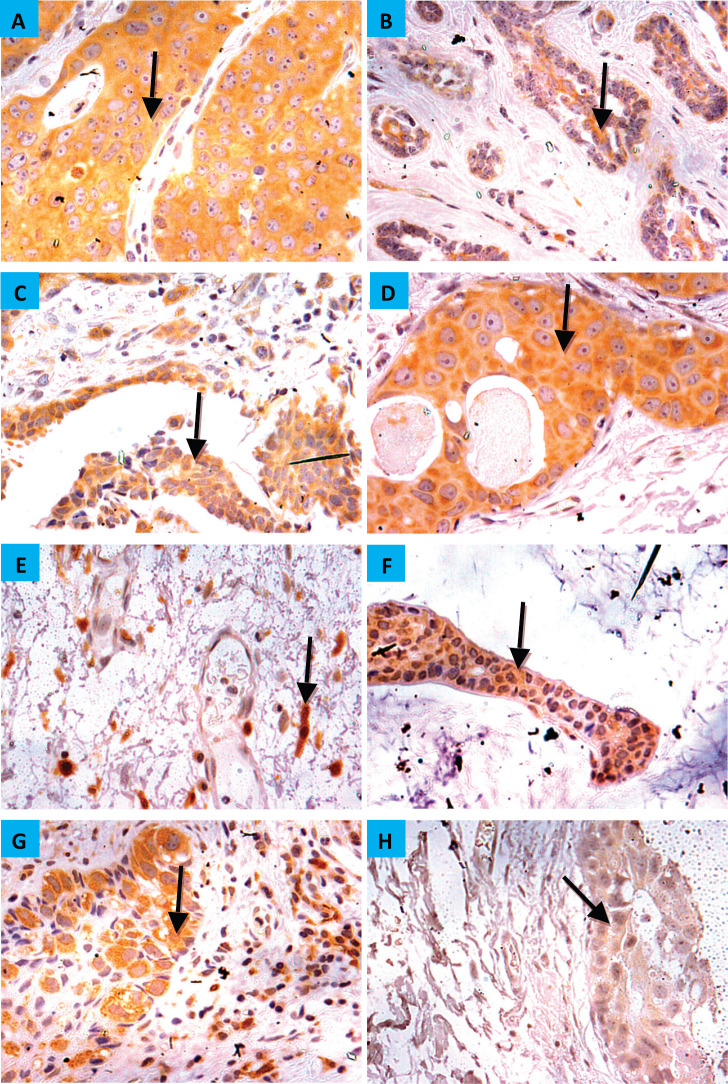
CYP4Z1 expression in breast carcinoma. Tumours were classified on the bases of histological type. (A) invasive ductal carcinoma, (b) invasive lobular carcinoma, (c) invasive papillary carcinoma, (d) intra-ductal carcinoma, (e) invasive apocrine carcinoma, (f) mucinous carcinoma, (g) Paget’s disease and (h) lobular carcinoma in situ. Magnification (×400).

The demographic and clinicopathologic features of breast tumours, stratified for CYP4Z1 positive and CYP4Z1 negative, are provided in Table 1. CYP4Z1 was differently expressed across the panel of breast tumours. In malignant tumours, 187 cases (82.0%) showed a moderate–intense expression, while others displayed a no to weak expression (41 cases, 18.0%). The normal tissues and benign tumours showed a no to weak expression. There was a significant relationship between CYP4Z1 expression and histological type of tumour (*p* < 0.05). CYP4Z1 was strongly expressed in 148 cases of invasive ductal carcinoma (83.1%), 13 cases of invasive lobular carcinomas (100%), 12 cases of intra-ductal carcinomas (85.7%), five cases of mucinous adenocarcinomas (83.8%), two cases of invasive papillary carcinomas (100%), 2 lobular carcinoma *in situ* (100%) and two cases of invasive apocrine carcinoma (100%) (Figure 3). According to histological grade, a significant relationship (*p* < 0.05) was found with CYP4Z1 expression, where most CYP4Z1 positive cases were either grade II (44.2%) or grade III (53%). Interestingly, the rate of CYP4Z1 expression tended to increase with the tumour grade.

The relationship between the CYP4Z1 expression and status of lymph node metastasis was also investigated. There was a significant association between CYP4Z1 expression and lymph node status (*p* < 0.05%). CYP4Z1 positive tumours (82%) had a higher rate of lymph node metastasis than CYP4Z1 negative tumours (18%). On the contrary, no statistical difference in CYP4Z1 expression was observed in relation to the age of patients. However, when the patients’ age was categorised into two groups (≤50y and >50y), CYP4Z1 had a higher rate of expression in the group of patients below 50 years (58.3%) than the group of patients above 50 years (41.7%). Although there was no statistical difference between CYP4Z1 expression and histological stage, the trend of CYP4Z1 expression tended to be higher in stage 2 (87.6%) and stage 3 (89.2%) of the disease than in stage 1 (84.2%).

#### Association between CYP4Z1 expression and immunohistochemical markers

To determine the accurate relationship between CYP4Z1 expression and different immunohistochemical markers, samples were categorised according to the status of CYP4Z1 (CYP4Z1-negative and CYP4Z1-positive). CYP4Z1 expression was studied in association with AR, ER, EGFR, PR, HER2, P53 and Ki-67 separately. It was found that CYP4Z1-positive patients showed a higher rate of AR expression when compared with CYP4Z1-negative patients (85.6% versus 14.4%), although the relationship was not statistically significant. Likewise, there was a proportional relationship between CYP4Z1 expression and ER expression. No statistical difference was detected, despite the fact that CYP4Z1-positive patients showed higher rates of ER expression compared to CYP4Z1-negative patients (83.7% versus 16.3%). Moreover, no significant difference in CYP4Z1 expression was seen between EGFR-positive patients and EGFR-negative patients (83.2% versus 77.3%).

The ratio of PR expression was higher in CYP4Z1-positive patients than in CYP4Z1-negative patients (83.8% versus 16.2%). However, no statistical difference was detected. Interestingly, there was a significant difference in CYP4Z1 expression in relation to HER2 and P53 status (*p* < 0.05%). A significant number of CYP4Z1-positive patients were associated with positive HER2 expression (82.9% of CYP4Z1-positive vs 17.1% of CYP4Z1-negative). The P53 expression tended to have higher rates of expression in CYP4Z1-positive patients than CYP4Z1-negative patients (84.1% versus 15.9%). Comparisons of Ki-67 expressions between CYP4Z1-positive and negative patients showed significantly higher rates in the CYP4Z1-positive patients (84.7% versus 15.3%, *p* < 0.05%) (Table 2).

## Discussion

The present study has examined the expression of CYP4Z1 in a panel of different human tissues, including most of the common benign, malignant and metastatic tumours. This confirms the hypothesis that CYP4Z1 is overexpressed in tumours and is, as such, a valid biomarker or drug target for the development of novel therapeutic strategies. The data presented support and extend previous investigations in which the overexpression of CYP4Z1 was displayed in many human tumours [11, 12, 14, 15]. In this study, for the first time, we present convincing evidence of CYP4Z1 expression in many tumours other than cancers of breast, ovary, prostate and colon, and we demonstrate the general absence of expression in the corresponding normal tissues. CYP4Z1 was found to be expressed in carcinomas of adrenal cortex, squamous cell of oesophagus, lung and cervix, as well as seminoma, astrocytoma, melanoma and lastly endometrial adenocarcinoma.

Our observations support previous studies where the CYP4Z1 expression profile was assessed in breast, ovary, colon and prostate cancers. CYP4Z1 was highly expressed in all tumours examined when compared to corresponding normal tissues [14–17]. Moreover, some degree of immunoreactivity was observed for CYP4Z1 in metastatic cancer tissues when compared to primary tumours [15, 16]. Importantly, CYP4Z1 expression was considered an independent prognostic biomarker for prostate and ovarian cancers [15, 17]. This validates the results of our screening. Further studies, using larger sample sizes, are needed for better screening of CYP4Z1 expression in tumours.

Despite the growing evidence for the contribution of CYP4Z1 to tumour malignancy, this is the first study that solely investigated the CYP4Z1 expression in breast cancers and correlated its expression with clinicopathological features and key molecules playing an important role in breast cancer pathogenesis. Here, CYP4Z1 was found to be highly expressed in malignant tumours when compared to corresponding normal tissues and benign tumours. This was consistent with previous studies showing selective expression of CYP4Z1 in mammary glands and breast cancers [11, 14].

Although the sample size of the study was small, some significant associations between CYP4Z1 expression and clinicopathological features were elaborated. CYP4Z1 expression was significantly associated with the histological grade and status of lymph node metastasis. CYP4Z1 expression was strongly associated with increasing tumour histological grades. Similarly, Rieger *et al* [14] demonstrated this kind of association where a strong CYP4Z1 expression was seen in high-grade tumours. The pattern of CYP4Z1 expression and involvement of lymph node metastasis in breast cancer has not been studied previously. Despite the small number of patients with lymph node metastasis, a high rate of lymph node metastasis was seen in CYP4Z1-positive patients. Unfortunately, no significant associations were possible between CYP4Z1 expression, age of patient and histological stage. However, a clear trend of high CYP4Z1 expression was apparent in young patients (below 50 years) and advanced stage of disease. These observations reflect the role of CYP4Z1 in the malignancy of breast cancer.

While there have been limited studies evaluating CYP4Z1 expression in breast cancer [14], none has attempted to explore the relationship between the expression of CYP4Z1 and key molecules involved in breast cancer pathogenesis. In the era of molecular oncology, biomarkers such as AR, ER, PR, EGFR, HER2, P53 and Ki-67 may provide valuable information and deeper insights into the pathogenesis of breast cancer. Thus, in this study, these biomarkers were selected based on their well-known prognostic value to assess the value of CYP4Z1 as a biomarker or drug target for the development of novel therapies.

It is well known that steroid hormones (AR, ER and PR) play an important role in controlling the growth and differentiation of normal and cancerous breast cells [22]. Their role as prognostic factors in breast cancer is well documented, but the significance of CYP4Z1 is still unclear. Here, higher rates of steroid hormone expressions were seen in CYP4Z1-positive patients compared to CYP4Z1-negative patients. No robust, statistically significant associations were possible. Similar results were obtained by Murray *et al* [14], who could not find any correlations between CYP4Z1 expression and the status of ER and PR in breast cancer. One of the important molecular predictors of prognosis in breast cancer is EGFR. EGFR overexpression is thought to be associated with large tumour size and poor clinical outcomes [23]. Our results showed comparable CYP4Z1 expression in EGFR-positive and EGFR-negative patients.

HER2 is one of the human epidermal growth factor receptors. It governs the proliferation, growth and survival of tumour cells and its over-expression is related to poor clinical prognosis in breast cancer [24]. In this study, there was a significant association between expressions of CYP4Z1 and HER2. The CYP4Z1 positivity rate was more than 80% in HER2-positive patients, indicating that CYP4Z1 plays an important role in the clinical prognosis of breast cancer. Likewise, P53 positivity rates were significantly different in the CYP4Z1 expression subtype groups, ranging from 84.1% in CYP4Z1-positive patients to 15.9% in CYP4Z1-negative patients. This abnormal P53 expression in breast cancer was associated with more aggressive tumour features, a higher tumour grade and poor prognosis [25]. One more important significant association with CYP4Z1 expression was the Ki-67 expression. Ki-67 is a proliferation biomarker which is considered to be a prognostic factor for the management of breast cancer [26]. Higher Ki-67 labelling was seen in CYP4Z1-positive patients than in CYP4Z1-negative patients. These findings therefore highlight that the potential role of CYP4Z1 expression in breast tumour genesis is complex. Further investigations are needed in this regard.

In an attempt to investigate the mechanistic role of CYP4Z1 in tumour progression, several studies have demonstrated an overexpression of CYP4Z1 in cell line models and human tumour xenografts [19, 27–29]. CYP4Z1 expression was shown to be highly induced in breast cancer cells by treatment with dexamethasone or progesterone [19, 28]. This CYP4Z1 expression was translocated to the plasma membrane of breast cancer cells, while nothing displayed on the surface of normal breast cells [28, 30]. Additionally, CYP4Z1 overexpression enhanced tumour angiogenesis, growth and spread of breast cancer cells in both *in vitro* and *in vivo* models [19, 27, 29]. This was due to an increase in the production of vascular endothelial growth factor-A and a decrease in tissue inhibitor of metalloproteinase-2 expression via ERK1/2 and PI3K/Akt activation [19, 27]. It is notable that all these effects were accompanied by a decrease in levels of myristic and lauric acids, as well as increased production of 20-HETE [19]. This change in fatty acid levels was concordant with the hypothesis that CYP4Z1 is responsible for the metabolism of myristic and lauric acids [18], as well as conversion of arachidonic acid to 20-HETE, resulting in tumour angiogenesis and growth of breast cancer [19, 29]. A recent functional analysis, using a recombinant enzyme system, contradicted such a scenario, where 20-HETE metabolites were undetectable in the CYP4Z1 reaction with arachidonic acid (20). Further studies looking deeply into the mechanistic role of CYP4Z1 in tumour progression are also needed.

## Conclusion

The current study reveals, for the first time, an expression of CYP4Z1 in many different types of tumours with absence of the expression in corresponding normal tissues. By focusing on breast cancer, we also demonstrate for the first time the significant relationships between CYP4Z1 unique expression and clinicopathological features, as well as identify the key molecules involved in breast cancer pathogenesis. These results provide strong evidence in support of CYP4Z1 as a novel opportunity for the development of a new therapeutic strategy in the treatment of tumours expressing CYP4Z1.

## List of abbreviations

CYPs: Cytochromes P450
CYP4Z1: Cytochrome 4Z1
AR: Androgen receptor
EGFR: Epidermal growth factor receptor
ER: Oestrogen receptor
PR: Progesterone receptor
HER-2: Epidermal growth factor receptor 2
20-HETE: 20-hydroxyeicosatetraenoic acid

## Authors’ contributions

YMS and NSA participated in the clinical assessment of the enrolled subjects and the data collection, and conducted the data analysis and manuscript revisions. SS, JMS, NSA and MNN participated in the development of the study protocol and the study design, and conducted the data analysis, interpretation of the findings, manuscript writing and manuscript revisions. AS participated in the statistical analysis and manuscript revisions. HMR and OH participated in the statistical analysis and manuscript revisions. HMR and YMS participated in the study design, and conducted the data analysis, interpretation of the findings and manuscript revisions. All authors read and approved the final manuscript.,

## Funding

This research received no external funding.

## Conflicts of interest

The authors declare no conflict of interest.

## Ethics approval and consent to participate

The study was ethically approved by the Ethics Committee, Faculty of Medicine, Mutah University, Jordan.

## Consent for publication

These are archived, formalin-fixed, paraffin-embedded tissue blocks (4 μm thick) that were obtained from the surgical pathology files of King Hussin Medical Hospital, Royal Medical Services and King Abdullah University Hospital, Amman and Irbed, Jordan. The consent from each patient had been taken by the hospital.

## Availability of data and material

Data sharing not applicable to this article as no datasets were generated or analysed during the current study.

## Competing interests

The authors declare that they have no competing interests.

## Figures and Tables

**Table 1.** Association between CYP4Z1 expression and studied clinicopathological features.

Characteristic	CYP4Z1-positive *n* = 187	CYP4Z1-negative *n* = 41	*p* value
**Age (years)**			
≤50 (*n* = 135, 59.3%)	109 (58.3%)	26 (63.4%)	0.244
>50 (*n* = 93, 40.7%)	78 (41.7%)	15 (36.6%)	
**Histological type**			
Invasive ductal carcinoma (*n* = 178, 80.8%)	148 (83.1%)	30 (16.9%)	0.021
Invasive lobular carcinoma (*n* = 13, 5.9%)	13 (100%)	0 (0%)	
Intra-ductal carcinoma (*n* = 14, 6.4%)	12 (85.7%)	2 (14.3%)	
Mucinous adenocarcinoma (*n* = 6, 2.7%)	5 (83.3%)	1 (16.7%)	
Invasive papillary carcinoma (*n* = 2, 1%)	2 (100%)	0 (0%)	
Lobular carcinoma in situ (*n* = 2, 1%)	2 (100%)	0 (0%)	
Invasive apocrine carcinoma (*n* = 2, 1%)	2 (100%)	0 (0%)	
Others: (*n* = 3, 1.5%)	2 (66.6%)	1 (33.3%)	
**Histological grade**			
I (*n* = 16, 7%)	11(2.8%)	5 (12.2%)	0.04
II (*n* = 93, 40.7%)	80 (44.2%)	13 (31.7%)	
III (*n* = 119, 52.2%)	96 (53%)	23 (56.1%)	
**Lymph node metastasis**			
Yes (*n* = 61, 26.8%)	50 (82%)	11 (18.0%)	0.044
No (*n* = 158, 69.3%)	131 (82.9%)	27 (17.1%)	
NA (*n* = 9, 3.9%)			
**Histological stage**			
1 (*n* = 38, 16.7%)	32 (84.2%)	6 (15.8%)	0.447
2 (*n* = 144, 63.2%)	116 (87.6%)	28 (12.4%)	
3 (*n* = 37, 16.2%)	33 (89.2%)	4 (10.8%)	
NA (n=9, 3.9%)			

**Table 2.** Association between CYP4Z1 expression and studied immunohistochemical markers.

Immunohistochemical marker	CYP4Z1-positive *n* = 187	CYP4Z1-negative *n* = 41	*p* value
**AR**			
Positive (*n* = 125, 54.8%)	107 (85.4%)	18 (14.6%)	0.084
Negative (*n* = 103, 45.2%)	80 (77.7%)	23 (22.3%)	
**ER**			
Positive (*n* = 129, 56.7%)	108 (83.7%)	21 (16.3%)	0.227
Negative (*n* = 99, 43.3%)	79 (79.8%)	20 (20.2%)	
**PR**			
Positive (*n* = 105, 46%)	88 (83.8%)	17 (16.2%)	0.317
Negative (*n* = 123, 54%)	99 (80.5%)	24 (19.5%)	
**EGFR**			
Positive (*n* = 44, 19.3%)	34 (77.3%)	10 (22.7%)	0.239
Negative (*n* = 184, 80.7%)	153 (83.2%)	31 (16.8%)	
**HER2**			
Positive (*n* = 164, 71.9%)	136 (82.9%)	28 (17.1%)	0.002
Negative (*n* = 64, 29.1%)	51 (79.7%)	13 (20.3%)	
**P53**			
Positive (*n* = 157, 68.8%)	132 (84.1%)	25 (15.9%)	0.02
Negative (*n* = 71, 31.2%)	55 (77.5%)	16 (22.5%)	
**Ki-67**			
<14% (*n* = 111, 48.7%)	93 (79.5%)	24 (20.5%)	0.033
>14% (*n* = 117, 51.3%)	94 (84.7%)	17 (15.3%)	
